# Sequence-specific procedural learning deficits in children with specific language impairment

**DOI:** 10.1111/desc.12125

**Published:** 2014-01-11

**Authors:** Hsinjen Julie Hsu, Dorothy VM Bishop

**Affiliations:** 1 Graduate Institute of Audiology and Speech Therapy, National Kaohsiung Normal UniversityTaiwan; 2 Department of Experimental Psychology, University of OxfordUK

## Abstract

This study tested the procedural deficit hypothesis of specific language impairment (SLI) by comparing children’s performance in two motor procedural learning tasks and an implicit verbal sequence learning task. Participants were 7- to 11-year-old children with SLI (n = 48), typically developing age-matched children (n = 20) and younger typically developing children matched for receptive grammar (n = 28). In a serial reaction time task, the children with SLI performed at the same level as the grammar-matched children, but poorer than age-matched controls in learning motor sequences. When tested with a motor procedural learning task that did not involve learning sequential relationships between discrete elements (i.e. pursuit rotor), the children with SLI performed comparably with age-matched children and better than younger grammar-matched controls. In addition, poor implicit learning of word sequences in a verbal memory task (the Hebb effect) was found in the children with SLI. Together, these findings suggest that SLI might be characterized by deficits in learning sequence-specific information, rather than generally weak procedural learning.

## Introduction

In recent years a distinction has been made between two knowledge systems, which are both functionally and anatomically separate. Declarative memory, primarily supported by the medial temporal lobes, is responsible for storage and conscious recall or recognition of facts and events. Procedural memory, which is dependent on frontal/basal ganglia structures, is involved in the implicit learning of motor skills or cognitive routines, and is typically achieved via repeated exposure to activities constrained by rules or patterns. Historically, the distinction between procedural and declarative memory systems emerged from studies of neuropsychological patients, such as those with amnesia, who performed normally on a wide variety of procedural learning tasks (e.g. mirror drawing, pursuit rotor, artificial grammar learning, probabilistic classification learning) but were impaired on tests of declarative memory (Brooks & Baddeley, 1976; Cohen & Squire, 1980; Gordon, 1988; Graf, Squire & Mandler, 1984). In the years since these learning systems were first described, there has been some debate over whether the distinction between them is as clear-cut as this account implies; for instance, medial temporal lobe structures have been implicated in classic procedural tasks such as motor sequence learning (Schendan, Searl, Melrose & Stern, 2003) and probabilistic classification (Poldrack, Prabakharan, Seger & Gabrieli, 1999; Poldrack, Clark, Pare-Blagoev, Shohamy, Creso Moyano, Myers & Gluck, 2001). Nevertheless, dissociations between types of memory problem in neuropsychological patients emphasize that memory is not unitary, and there has been interest in identifying the types of skills that can be dissociated.

Ullman adopted the distinction between two forms of memory in formulating a declarative-procedural model of language (Ullman, 2001, 2004). This postulates that the procedural memory system is important for learning and the use of rule-governed aspects of grammar (syntax, morphology and phonology), and contrasts with a declarative learning system that is involved in acquisition of vocabulary and more general semantic knowledge. Ullman and Pierpont (2005) went on to interpret a common developmental disorder, specific language impairment (SLI), within this framework as a procedural learning deficit. The procedural deficit hypothesis attempts to explain a common language profile seen in many children with SLI, i.e. grammar is disproportionately impaired while vocabulary is relatively spared. Ullman and Pierpont argued that SLI is not specific to language, but involves all aspects of learning depending on frontal/basal ganglia circuits. They therefore predicted that children with SLI should show broader impairments on nonlinguistic tasks that involve procedural learning.

Several recent studies tested the procedural deficit hypothesis of SLI using the serial reaction time (SRT) task. In a typical SRT task, participants respond to a visual stimulus that appears in one of four locations on a computer screen by pressing a corresponding button on a four-button response panel. On some blocks the visual stimuli would follow a predetermined repeating sequence of locations (pattern phase), whereas on others the visual stimulus would appear in random locations from trial to trial (random phase). Sequence learning in such tasks is indicated by a decrease in reaction times (RTs) during pattern phases and a re-bound in RTs when the task proceeds from a pattern phase (i.e. after learning) to a subsequent random phase.

Tomblin, Mainela-Arnold and Zhang (2007) were the first to examine SRT learning in individuals with SLI. They compared the performance of 15-year-old adolescents with SLI to typically developing age-matched controls using an SRT task composed of a random phase, followed by a pattern phase and then a second random phase. Although both groups showed a decrease in RTs during the pattern phases of the task, the adolescents with SLI showed a slower learning rate than the controls. In a further analysis, they re-grouped their participants in terms of their vocabulary and grammar abilities to explore the association between SRT learning and language abilities. When the participants were re-grouped into a grammar-impaired group and a normal-grammar group, the results were very similar to the original contrast between SLI and age-matched controls: the grammar-impaired group exhibited slower learning rate than the normal-grammar group during the pattern phases. However, when the participants were re-grouped into a normal-vocabulary group and a poor-vocabulary group, group differences in learning rates disappeared. These findings supported the prediction that deficits in SLI are not limited to the verbal domain but also occur in procedural learning of non-verbal skills. In addition, poor procedural learning of motor skills is specifically associated with grammatical deficits.

Poor SRT learning in SLI has been reported by Lum, Gelgec and Conti-Ramsden (2010), who used a slightly different SRT task and focused on changes in RT when the task shifted from the pattern phase to a subsequent random phase. They found a larger re-bound in RTs for control children than children with SLI, suggesting poorer sequence learning in the children with SLI. Subsequently, Lum, Conti-Ramsden, Page and Ullman (2011) investigated working memory, declarative memory and procedural memory in 10-year-old children with SLI and typically developing control children. Again, children with SLI were impaired at SRT learning, even when composite measures of working memory were held constant. In a recent study, Mayor-Dubois, Zesiger, Van der Linden and Roulet-Perez (2012) examined SRT learning in a group of French-speaking children with SLI and found that, although the overall analysis indicated that they learned a repeated sequence as well as control children, more fine-grained analysis suggested that their learning was slower than control children, even after excluding any child who had motor coordination difficulty. Gabriel and colleagues (Gabriel, Maillart, Guillaume, Stefaniak & Meulemans, 2011; Gabriel, Stefaniak, Maillart, Schmitz & Meulemans, 2012) conducted two studies on SRT learning in SLI where they found no evidence of procedural learning deficits. However, Gabriel, Maillart, Stefaniak, Lejeune, Desmottes and Meulemans (2013) found that when they used more complex sequences, they could replicate findings of deficient procedural learning. Finally, Hedenius and colleagues (Hedenius, Persson, Tremblay, Adi-Japha, Verissimo, Dye, Alm, Jennische, Tomblin & Ullman, 2011) used an alternating SRT task to compare probabilistic sequence learning and consolidation in children with SLI and typically developing children by comparing their performance in an initial learning session and three days later. Similar levels of performance were found between the two groups in an initial analysis. However, when the children were re-grouped in terms of their grammar abilities, a clear pattern emerged: whereas the grammar-impaired and normal-grammar groups showed initial learning of probabilistic sequences, only the normal-grammar group showed consolidation of the knowledge acquired from the initial session. These results extended the previous findings by showing deficits in long-term learning of motor procedural skills in SLI.

Procedural memory or learning is an aggregate of many skills and is conventionally tested with several paradigmatic tasks such as mirror drawing, pursuit rotor, and probabilistic categorization. One implication of the procedural deficit hypothesis is that children with SLI should show a general difficulty with all such tasks. However, studies on other clinical populations have found dissociations among procedural tasks. For instance, Harrington, Haaland, Yoe and Marder (1990) reported dissociations between mirror drawing and pursuit rotor performance in some patients with Parkinson’s Disease. Dissociations among procedural learning tasks have also been found in children with dyslexia (Gabay, Schiff & Vakil, 2012). Given that most evidence for poor procedural learning in SLI comes from studies using SRT tasks (cf. Kemeny & Lukacs, 2010), research using other procedural learning paradigms is needed to further evaluate the procedural deficit hypothesis.

Tomblin and colleagues (2007) suggested that the association between SRT learning and grammatical abilities might lie in individual differences in some very basic general purpose mechanisms that are involved in sequence learning. Indeed, processing and learning of serial order plays a crucial role in many aspects of human behaviour. Language, in particular, involves learning and processing of complex sequential structures, such as the sequence of phonemes that form a word, sequence of words that form a phrase, and word order, adjacent dependencies (e.g. determiners such as ‘a’ and ‘the’ always occur before a noun phrase) and nonadjacent relationships (e.g. *is* V-*ing*). Poor sequence learning in SLI has also been demonstrated in artificial language learning tasks (Evans, Saffran & Robe-Torres, 2009; Mayor-Dubois *et al*., 2012; Plante, Gomez & Gerken, 2002).

It is clear that sequence learning is a key feature in SRT tasks. However, not all commonly used motor procedural learning tasks focus on sequence learning. For instance, pursuit rotor tasks have been used for many years to test motor procedural learning in typical and atypical populations (Grafton, Mazziotta, Presty, Friston, Frackowiak & Phelpsis, 1992; Grafton, Woods & Tyszka, 1994; Harrington *et al*., 1990; Kumari, Gray, Honey, Soni, Bullmore, Williams, Ng, Vythelingum, Simmons, Suckling, Corr & Sharma, 2002; Sarazin, Deweer, Merkl, Poser, Pillon & Dubois, 2002; Schmidtke, Manner, Kaufmann & Schmolck, 2002). The tasks involve participants holding a wand to track the circular movement of a turntable-like platter by keeping the tip of the wand on a metal spot on the platter. Performance on such tasks requires learning appropriate adjustments of hand movements according to upcoming visual input (i.e. hand–eye coordination). Unlike SRT tasks, there is no sequential pattern of discrete elements embedded in the task. Given previous findings of poor SRT and artificial grammar learning in SLI, it is of interest to ask whether deficits in SLI affect all procedural tasks, or whether difficulties are specific to procedural tasks that involve sequence learning.

In the current study, we compared children with SLI and typically developing children using a novel verbal test of implicit sequential learning, i.e. Hebb learning, and two motor procedural learning tasks, one involving sequence learning (SRT task) and one that did not (pursuit rotor task).

The goals of the current study were threefold:

1. We considered whether children with SLI are impaired in verbal sequence learning in a short-term memory task (Hebb, 1961). To show the Hebb effect, sequences of words are administered for immediate recall, with some sequences being repeated. The participant is not told about the repeated sequences and may remain unaware of them, but nevertheless, recall is better for repeated than for novel sequences. This task has two advantages over the artificial grammar learning tasks that have been used in this area. First, it is much simpler and less taxing than artificial grammar learning, and uses familiar verbal materials. Second, the task can be adjusted to control for the level of immediate verbal memory, giving a relatively pure measure of implicit long-term learning of sequences. This is important because poor verbal short-term memory is a well-known characteristic of SLI (see Gathercole, 2006, for a review). Unless it is controlled for, we cannot know whether poor learning is due to poor immediate memory for words (or nonwords in the case of artificial grammar), or difficulties in forming longer-term representations of sequences.
  As far as we are aware, Hebbian learning has not previously been assessed in children with SLI, although Szmalec, Loncke, Page & Duyck, (2011) demonstrated reduced Hebb learning in adults with dyslexia, a condition that often co-occurs with SLI (Bishop & Snowling, 2004).
2. Previous studies showing poor motor procedural learning in SLI are largely based on performance in SRT tasks. Here, we compared performance in two motor procedural learning tasks, one that involved learning of a sequenced pattern (i.e. the SRT task) and one that focused on sensory-motor learning (i.e. the pursuit rotor task). If children with SLI have a global deficit in procedural learning, we should find a similar pattern of performance between groups in both tasks. However, if poor sequence learning is the key deficit in SLI, we would expect to see superior performance in the pursuit rotor task compared with the SRT task. In addition, Hedenius and colleagues (2011) have found poor consolidation of SRT learning in children with poor grammar. In the current study, we tested whether the same would apply to a pursuit rotor task.
3. For both verbal and nonverbal learning tasks, we compared children with SLI with an age-matched control group, but in addition we tested a ‘grammar-matched’ group of younger typically developing children. This allowed us to see whether learning ability was consistent with language level in those with SLI.

## Methods

### Participants

Three groups of children were recruited: (a) 7- to 11 year-old children with SLI (SLI, *N *=* *48); (b) typically developing children matched for chronological age (A-Match, *N *=* *20); and (c) younger typically developing children matched for receptive grammar (G-Match, *N *=* *28). Here, we report data from all three groups in an SRT task and a pursuit rotor task. For the Hebb learning task, data were collected from the two control groups and a subset of the children with SLI (*N *=* *28).

All of the children with SLI were recruited from special schools for children with language impairment or support units in mainstream schools. Children were included if they met all of the following screening criteria: (1) performed below 1 *SD* on at least two out of the following six standardized tests: the British Picture Vocabulary Scales II (BPVS II; Dunn, Dunn, Whetton & Burley, 1997), Test for Reception of Grammar-Electronic (TROG-E; Bishop, 2005), the comprehension subtest of the Expression, Reception and Recall of Narrative Instrument (ERRNI; Bishop, 2004), repetition of nonsense words subtest of the Developmental Neuropsychological Assessment (NEPSY; Korkman, Kirk & Kemp, 1998), and syntactic formulation and naming subtests of the Assessment of Comprehension and Expression 6–11 (ACE 6–11; Adams, Cooke, Crutchley, Hesketh & Reeves, 2001); (2) had a nonverbal IQ of 85 or above, as measured with the Raven’s Coloured Progressive Matrices (RCPM; Raven, 1995); (3) were able to hear a pure tone of 20 dB or less in the better ear, at 500, 1000, 2000 and 4000 Hz; (4) had English as their native language; and (5) did not have a diagnosis of other developmental disorders such as autism, Down Syndrome or Williams Syndrome.

The same screening tests were used to confirm language status for each child in the grammar- and age-matched groups. Both typically developing groups met the same criteria for nonverbal IQ, hearing and native language and did not score below 1 *SD* on more than one of the six standardized language tests or have a history of speech, language, social or psychological impairments. Descriptive information on the participants is given in Table 1. Group differences in nonverbal IQ measured with RCPM were not significant (*F*(2, 93) = 1.26, *p *=* *.29).

**Table 1:** Group summary statistics of the grammar-match group, the age-matched group and the SLI group

	G-Match	A-Match	SLI
*N*	28	20	48
Age (year; month)	5;81 (.9)	8;93 (.9)	8;8 (1.3)
Standard scores			
RCPM	105.2 (8.5)	105.8 (11.4)	101.9 (12.1)
TROG-E	102.3 (14.0)	97.6 (10.3)	74.3 (12.8)
ERRNI-Com	103.0 (14.6)	101.9 (12.6)	84.7 (15.7)
BPVS	108.3 (9.5)	102.6 (7.6)	87.7 (11.5)
ACE Naming	101.1 (8.0)	98.8 (11.9)	82.5 (11.6)
ACE Syntactic	105.5 (14.2)	98.5 (15.1)	81.8 (11.2)
NEPSY NWR	104.3 (15.0)	101.8 (15.8)	84.7 (15.6)
Raw scores			
RCPM	17.9 (4.6)	26.3 (4.7)	24.2 (4.9)
TROG-E	9.8 (3.1)	14.6 (2.4)	8.9 (3.8)
ERRNI-Com	8.4 (3.1)	12.9 (2.6)	8.9 (3.6)
BPVS	67.1 (13.0)	92.6 (9.2)	71.9 (16.6)
ACE Naming	10.0 (2.8)	16.3 (3.0)	11.1 (3.9)
ACE Syntactic	17.8 (5.93)	24.1 (5.13)	15.9 (5.86)
NEPSY NWR	27.0 (7.60)	32.2 (8.48)	23.0 (8.64)

Each child in the grammar-matched group had a TROG-E raw score (i.e. number of blocks passed) within three blocks of one of the children in the SLI group. Group differences on TROG-E raw score were not significant between the SLI and the grammar-matched group.

### Testing schedule

Children in this study also took part in a training study (to be described elsewhere), for which the SLI cases were randomly assigned to one of two subgroups. Children in the first SLI subgroup and the two control groups were seen over a 2-week period, during which they completed two screening sessions (language, hearing, nonverbal IQ), followed by four sessions of language training and a post-test session. The SRT and pursuit rotor tasks were given in the first two sessions. The pursuit rotor task was administered again in the post-test session to examine retention. The Hebb learning task was given in the third or the fourth language training session. Children in the second SLI subgroup followed the same schedule, except that they did not do the training sessions and were not given the Hebb learning task. Preliminary comparisons revealed no differences between the two SLI subgroups on the tests reported here, so they are treated together for the current study.

### Tasks

#### Hebb repetition learning task

A Hebb repetition learning task was created using C++ Builder to examine verbal sequence learning in children. On each trial, the overt task performed by the child was to remember a sequence of named items. However, some of the sequences recurred on subsequent trials, allowing us to assess implicit learning of the repeated sequence (Hebb effect). The visual layout of the task involved a little boy swimming in the sea with fishing nets in his hand. The fishing nets were displayed horizontally and were labelled with numbers from left to right to signal their order. For instance, the leftmost fishing net was labelled ‘1’ and the second to the left was labelled ‘2’ and so on. During the task, children listened to lists of familiar words and saw pictures of the words appear in random order at the bottom of the computer screen immediately after the auditory presentation of the words. They then clicked the pictures in the same order as they heard the corresponding words. Once the first picture was clicked, it moved automatically to the first fishing net, and so on for the rest of the pictures until all the pictures were clicked. Responses were automatically recorded by the program. For each trial, feedback was given with a football moving up a vertical bar on the right-hand side of the screen to indicate how accurate the responses were (e.g. ball jumps higher to indicate high accuracy).

The task was composed of two parts. In the first part, we measured each child’s word span in order to decide the length of the lists to use later in the Hebb repetition learning task. This was to prevent floor or ceiling effects that might arise if a fixed number of words were used for all children. In addition, this provided a control over individual differences in short-term memory and therefore allowed for a more direct investigation of sequence learning. For the word span measure, the task began with a list of three words and moved up one level (i.e. four words) each time a correct response was provided. When an incorrect response was given, a second attempt at the same list length was provided. The program stopped automatically when two errors were made for a given list level. Children’s word span was determined by the longest length at which they were able to give at least one correct response.

In the second part of the task, children performed the Hebb repetition learning task in which they listened to a total of 13 word lists and clicked pictures of the words in the same order. For each child, list length was determined by adding one word to the child’s word span. Of the 13 word lists, five were *repeated* lists which always contained the same words in the same sequential order (e.g. dog bus sock comb), whereas the other eight were *non-repeated* lists which contained different words that never occurred in any other trials. The repeated and non-repeated lists were interleaved in a way such that every third list starting from the first list was a repeated list (i.e. 1st, 4th, 7th, 10th, and 13th lists). The words used to form the lists were all nouns within the first two years of developmental lexicon in typically developing children (Hamilton, Plunkett & Schafer, 2000). Because our focus was on learning the sequence of the words rather than the words *per se*, highly familiar words were used. An example of task materials is provided in the Appendix.

#### Serial reaction time (SRT)

The SRT task developed by Tomblin *et al*. (2007) was adopted in this study. Children sat in front of a laptop computer and saw four empty squares horizontally arranged on the computer screen. A response box with four horizontally arranged buttons that was connected to the computer was placed in front of children. Each button from left to right on the response box corresponded to an empty square, from left to right, on the computer screen. Children first rested their index and middle fingers of both hands on the buttons, and were asked to press the corresponding button, as accurately and as quickly as possible, each time they saw a green creature appear in one square on the screen. The task was composed of four phases: a random phase, followed by two pattern phases and a final random phase. In the first random phase, the green creature appeared randomly for 100 trials. In the two pattern phases, the location of the green creature followed a predetermined sequence (1-3-2-4-4-2-3-4-2-4) unbeknown to children for 100 trials per phase. In the final random phase, the green creature again appeared randomly for 100 trials.

The control of the image presentation and recording of responses (accuracy, reaction time) were accomplished by E-Prime Software. For each trial, the image with all four empty boxes appeared on the screen for 500 ms, followed by the image of the green creature in a box, which would disappear as soon as a button was pressed. The task automatically proceeded to the next trial once a response was given. Before the task began, a series of practice trials with feedback was provided to all children to learn the association of the buttons with the location of the squares on the screen. Between each phase, children were given a short break followed by four ‘warm-up’ trials before the next phase began. The task lasted for 12–15 minutes. Learning of the sequence would be indicated by a decrease in RTs during the pattern phase and a re-bound in RTs from the pattern phase to the subsequent random phase.

#### Pursuit rotor task

A computerized pursuit rotor task was used to examine procedural learning of motor skills (Life Science Associates, Inc., Bayport, New York). In this task, children saw a red dot (1.5 cm in diameter) moving clockwise on a computer screen. They moved a stylus pen on a drawing board to maintain contact with the computer mouse cursor with the red dot. The task was composed of one practice trial and 15 test trials. Each trial contained five rotations at a speed of 22.5 revolutions per min. The dependent measure of the task was the percentage of time that the mouse cursor was kept on the red dot, which was automatically recorded by the program.

Before the task began, children were given a few practices with the stylus pen by moving the pen on the drawing board and observing the corresponding movement of the mouse cursor on the computer screen. The red dot then appeared in the top centre location on the screen, and children moved the stylus pen to keep the mouse cursor on the top of the red dot to get ready for the task. A timer, which then appeared next to the red dot, started to count down from 20 to 0 s to signal the beginning of each trial. Children were told to move the stylus pen to maintain contact with the red dot once the timer counted down to 0. To keep children engaged, the red dot made a beeping sound whenever the mouse cursor was on the target. At the end of each trial, the red dot stopped in the starting location (i.e. top centre of the screen). The timer counted down 20 s again (i.e. rest interval) to signal the beginning of the next trial. The same task was administered again 5–7 days after the first session to examine retention of the skills learned from the previous session.

## Results

### Hebb repetition learning task

For the Hebb repetition learning task, data were collected from the two typically developing groups and 28 of the children with SLI. Children’s word span was first measured to obtain baseline performance for the Hebb repetition learning task. A significantly higher word span was found in the age-matched group than the SLI and the grammar-matched groups (A-Match: mean = 4.90, *SD* = .79; SLI: mean = 3.96, *SD* = 1.07; G-Match: mean = 4.11, *SD* = .88; A-Match vs. SLI: *p *=* *.001; A-Match vs. G-Match: *p *=* *.005). The group difference in word span between the SLI and the grammar-matched groups was not significant (SLI vs. G-Match: mean difference = .14; *p *=* *.57).

As noted above, list length for each child was determined by adding one word to the child’s word span. For each trial, we calculated the percentage of items that were correctly recalled in their position. Figure1 shows the group mean accuracy for the repeated and non-repeated trials of the SLI, age- and grammar-matched groups.

**Figure 1:**
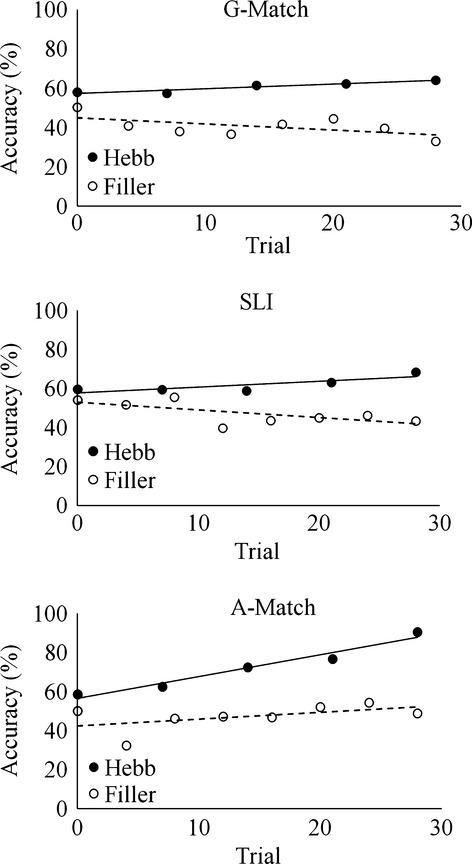
Mean accuracy for the grammar-matched, age-matched and SLI groups in the Hebb repetition trials. Regression lines were added to capture performance change over time for Hebb and filler trials.

Regression lines across trials were added to capture the effects of learning over time. Because list length was adjusted according to each individual’s memory capacity, all three groups exhibited similar levels of performance on the first trial (see Figure1). We first confirmed that both of the assumptions of normality and homogeneity of variance were met. To investigate group differences in the rates of learning word sequence, we compared the gradients of the repeated trials (i.e. learning rates) of the three groups by including the gradients of the non-repeated trials as a covariate in an analysis of covariance (ANCOVA). There was a significant effect of group (*F*(2, 76) = 3.68. *p *=* *.03, partial η^2^ = .09). Pair-wise comparisons indicated that the age-matched group showed a steeper learning rate of word sequences than the SLI and the grammar-matched group (A-Match vs. SLI: mean difference = 5.03, *p *=* *.04; A-Match vs. G-Match: mean difference = 6.45; *p *=* *.01). The group difference between the SLI and grammar-matched groups was not significant (mean difference = 1.42, *p *=* *.52). Together, these findings indicated poor implicit learning of word sequences in the children with SLI, with performance being comparable to that of younger typically developing children of similar language level.

We considered whether or not differences in the Hebb repetition learning task might be associated with individual variations in memory span. To this end, we examined the correlations between each individual’s word span and learning rate (i.e. gradient) of the repeated trials in the Hebb repetition learning task. The correlation coefficient between word span and gradient on repeated trials was non-significant (*r * = * *−.14, *p *=* *.25), indicating that the performance differences in the Hebb repetition learning task cannot be accounted for by individual differences in memory span.

### SRT task

#### Response accuracy

We first examined the percentage of correct button pressing in response to each visual stimulus. Table 2 shows mean response accuracy for each of the four phases in the grammar-matched, age-matched and SLI groups. Overall, high accuracy was observed for all three groups in each phase, although the grammar-matched group seemed to be less accurate than the other two groups. Response accuracy did not follow the normal distribution, with many data points clustered around the higher end of the 90–100% range. Given the violation of the normality assumption, we used a resampling method, bootstrap, to test our hypothesis (Wilcox, 2012). Unlike conventional ANOVA models, the bootstrap procedure does not assume normality or rely on the central limit theorem but instead uses the data at hand to estimate the sampling distribution of some statistic. The original data set is taken as the population from which random samples are repeatedly drawn (*bootstrap sample*) with replacement. Each of the bootstrap samples provides an estimate of the parameter of interest (e.g. mean) and relevant statistics (e.g. standard deviation), and these values are then aggregated into a bootstrap sampling distribution. This process is repeated a large number of times (e.g. 1000 times) to provide the required information on the variability of the estimator. For instance, the standard error is estimated from the standard deviation of the statistics derived from the bootstrap samples.

**Table 2:** Response accuracy (%) of the SLI, age-matched and grammar-matched groups in the SRT task. Standard deviations are reported in parentheses

	Random 1	Pattern 1	Pattern 2	Random 2
G-Mach	87.14 (13.04)	88.29 (9.88)	87.11 (10.23)	85.57 (13.80)
SLI	91.50 (6.90)	91.65 (6.24)	89.65 (8.78)	90.90 (6.32)
A-Match	93.05 (5.30)	92.80 (6.33)	91.85 (7.13)	91.65 (6.66)

Based on the method described in Wilcox (2012), the bootstrap-t method for testing hypotheses in a between-by-within design was adopted by using the R function *tsplitbt*. Our data involved three levels of between-subjects factor (SLI, G-Match, A-Match) and four levels of within-subjects factor (Random 1, Pattern 1, Pattern 2, Random 2). The number of bootstrap repetitions was set to 1000 with alpha level of .05.

The effect of group on accuracy was not significant (*Qa* = 2.08, *p *=* *.22). The effects of phase (*Qb* = 1.76, *p *=* *.13) and group × phase interaction (*Qab* = .20, *p *=* *.98) were also not significant.

#### Reaction time

Before we examined RT changes, we inspected the response accuracy of each child and excluded anyone whose overall accuracy was below 80%. This was to ensure reliable RT measures on the basis that children were able to press the right buttons. As a result, five children in the grammar-matched group and two children in the SLI group were excluded from RT analyses. We then collapsed trial by trial reaction times of each participant into the median response speed on correct trials across successive sets of 20 trials. Medians were used because the RT distributions are highly skewed and medians are more representative of the distribution central tendencies (see Tomblin *et al*., 2007). Figure2 presents the median RTs for all three groups.

**Figure 2:**
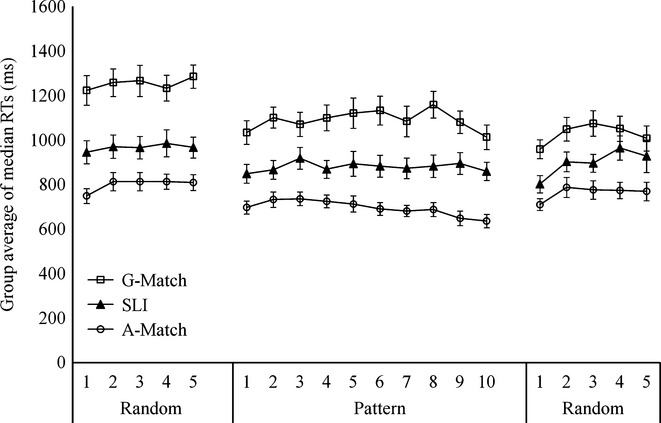
Group average of median RTs for the three groups in the SRT task. The error bars represent standard errors of the mean.

The major question associated with the SRT task was whether there is a group difference in the rate of learning sequence-specific information. This would be reflected in (a) decreases in RTs during the pattern phases and (b) a re-bound in RTs from the pattern to a subsequent random phase. To examine RT change during the pattern phases, we used the same methods as Tomblin *et al*. (2007) and compared group differences in learning rate during the pattern phase with growth curve analysis using the *nlme* package within R. The model contained a parameter for overall performance level (i.e. intercept) and three parameters for the shape of learning (linear slope, quadratic term, cubic term). Group identity was included as a fixed-effects predictor of the four parameters. A significant group effect on the shape of learning would indicate group differences in learning rates. Non-significant effects were taken out of the models. For the second analysis, we compared changes in RTs from the last block of the pattern phase to the first block of the subsequent random phase for all four groups. We calculated the gradient from the last block of the pattern phase to the first block of the subsequent random phase for each participant. Group differences in gradient were then statistically evaluated with one-way analysis of variance (ANOVA).

##### Learning during the pattern phases

The final model included an intercept, a linear slope, a quadratic term, a group effect on the intercept, and a group effect on the slope. This model showed that the SLI group was generally slower than the age-matched group (group difference in intercept: A-Match vs. SLI = 140.21, *t* = 2.13, *p *=* *.03) and significantly faster than the grammar-matched group (A-Match vs. G-Match = 177.79, *t* = 2.83, *p *=* *.01) at the first pattern block, which represents the intercept. The age-matched group was also significantly faster than the younger grammar-matched children at the beginning of the pattern phase (A-Match vs. G-Match: 349.51, *t* = 4.74, *p *<* *.00001). In order to investigate group differences in sequence learning rather than overall differences in RTs, we need to consider group effects on slope or higher-order terms. We found a significant group effect on the slope, due to a faster learning rate in the age-matched group than the SLI group and the grammar-matched group (A-Match vs. SLI: 9.84, *t* = 2.23, *p *=* *.03; A-Match vs. G-Match: 11.23, *t* = 2.24, *p *=* *.02). Differences in learning rate between the SLI and the grammar-matched groups were not significant.

##### Re-bound

In a second set of analyses, we examined changes in RTs when the task proceeded from the pattern phase to the subsequent random phase. As shown in Figure2, only the age-matched group showed a clear re-bound in RTs. The gradients from the last block of the pattern phase to the first block of the subsequent random phase were entered into a one-way ANOVA. There was a significant effect of group (*F*(2, 41.76) = 9.51. *p *<* *.0001), with a greater re-bound in RTs in the age-matched group than the other two groups (A-Match vs. SLI: mean difference = 132.29, *SE* = 39.62, *p *=* *.001; A-Match vs. G-Match: mean difference = 129.49, *SE* = 45.39, *p *=* *.01). Differences between the SLI and the younger grammar-matched groups were not significant. In general, our results were similar to previous findings of poor SRT learning in SLI, but we were able to show that comparable performance was seen in younger typically developing children who were functioning at a similar level in grammatical ability.

### Pursuit rotor task

Trial by trial performance in the pursuit rotor task was collapsed into three trials per block for a total of five blocks. Thus block 1 was the mean accuracy of trials 1–3, block 2 the mean accuracy of trials 4–6, and so on. Figure3 shows group means of each of the three groups on session 1 and session 2. Two sets of statistical analyses were conducted. First of all, we examined session 1 performance by comparing group differences in learning rates using growth curve analysis methods. In a second set of analyses, we inspected group difference in retention by comparing performance between the last block of session 1 and the first block of session 2.

**Figure 3:**
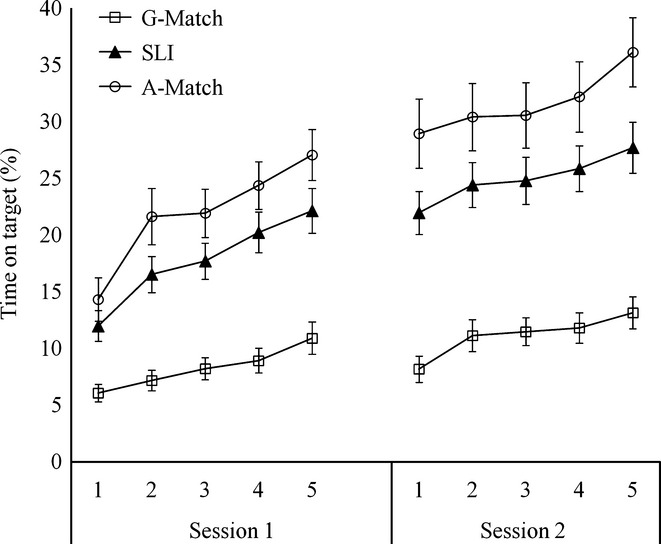
Group average of time on target (%) for the three groups in the pursuit rotor task. The error bars represent standard errors of the mean.

#### Session 1: acquisition of performance

First, we examined RT changes in session 1 using growth curve analysis methods.

The final model included an intercept, a slope, a quadratic term, a cubic term, a group effect on the intercept, a group effect on the slope and a group effect on the quadratic term. The mean accuracy of the grammar-matched group was significantly lower than SLI and the age-matched groups at the first block, which represents the intercept (G-Match vs. SLI: −6.19, *t * =  −3.17, *p *=* *.002; G-Match vs. A-Match: −9.08, *t * =  −3.76, *p *=* *.0001). The difference in intercept between the SLI group and the age-matched group was not significant. Our primary interest is in group differences in learning rates, which would be reflected by group effects on the shape of learning (linear slope, quadratic term, cubic term). There was a significant effect of group on linear acceleration, with the grammar-matched group showing a slower learning rate than both the SLI and the age-matched groups (G-Match vs. SLI: −2.78, *t * =  −3.35, *p *=* *.0009; G-Match vs. A-Match: −4.06, *t * =  −3.98, *p *=* *.0001). Unlike the SRT task, learning rate differences between the SLI group and the age-matched children were not significant in this task (SLI vs. A-Match: −1.29, *t * =  −1.39, *p *=* *.16). In addition to the group effect on linear slope, a significant group effect on the quadratic term was also observed. Again, the effect was mainly due to a difference in quadratic acceleration rate between the grammar-matched group and the other two groups (G-Match vs. SLI: .38, *t *=* *2.03, *p *=* *.04; G-Match vs. A-Match: .60, *t *=* *2.59, *p *=* *.01).

#### Session 2: maintenance of the acquired skill

The same task was repeated 5 to 7 days after the first session to examine retention of the skill acquired in the first session. The results are presented in Figure3. To evaluate group differences in maintaining the skills, the gradient between the last block of the first session to the first block of the second session of each individual was calculated and entered into a one-way ANOVA. There was a significant effect of group (*F*(2, 48.49) = 3.53, *p *=* *.037), with the grammar-matched group showing a shallower gradient than the age-matched group (mean difference = −4.61, *p *=* *.03). Group differences between the SLI and the age-matched groups or between the SLI and the grammar-matched group were not significant.

### Correlations between procedural and language tasks

Finally, we considered how the slope for learning on the Hebb repetition task related to the learning measures from the SRT task, and to grammar and vocabulary raw scores. Table 3 shows the bivariate correlation coefficients for the SLI and the grammar-matched children together. These two groups had similar memory spans and performed in the same range on the Hebb repetition learning task. The age-matched group was excluded to avoid spurious correlations that might arise from including individuals who performed at different levels on the experimental tasks. TROG raw scores were significantly correlated with BPVS raw scores, but not with performance in either the SRT task or the Hebb repetition task. The correlation between the SRT rebound and the learning slopes on the Hebb repetition task fell short of significance (*r *=* *.23, *p *=* *.09), but there was a significant correlation between the learning slopes on the SRT task and the Hebb repetition task (*r *=* *0.40, *p *=* *.002). Thus faster rates of decrease in RT during the pattern phase of the SRT task were associated with faster rates of learning in the Hebb repetition task, suggesting that individual differences in sequence learning may underpin performance in both tasks.

**Table 3:** Correlations between the learning slopes on the Hebb repetition learning task, TROG raw scores, BPVS raw scores, the learning slopes during the pattern phases of the SRT task and SRT rebound rate of the SLI and the grammar-matched children (N = 56)

Variables	1	2	3	4	5
1. Hebb repetition task	–				
2. TROG (raw scors)	.02	–			
3. BPVS (raw scores)	−.22	.51^**^	–		
4. SRT: pattern phase	−.40^**^	−.03	.03	–	
5. SRT: rebound	.23^a^	−.14	−.19	−.20	–

** *p *<* *.001 (2 tailed).

a marginally significant: *p *=* *.09.

## Discussion

We found poor implicit sequence learning by children with SLI in an implicit verbal learning task, even after controlling for limitations of verbal short-term memory. However, younger typically developing children showed a closely similar pattern on this task, confirming that the pattern of performance in SLI is not a qualitative deviation from typical development but an immaturity, though selectively affecting a specific type of learning.

We also considered motor procedural learning in SLI. Previous studies have largely used SRT tasks. We extended previous findings by including pursuit motor learning, a classic procedural task that does not involve learning a sequence of discrete elements. In the SRT task, the SLI group showed similar rates of motor sequence learning to younger grammar-matched children, but slower rates of learning than age-matched children. In addition, a larger re-bound in RTs was found for the age-matched children than the SLI and grammar-matched groups. Overall, these results are consistent with previous findings with SLI (Hedenius *et al*., 2011; Lum *et al*., 2010, 2011; Tomblin *et al*., 2007). However, a different pattern of performance was observed for pursuit rotor learning. Here, children with SLI performed comparably to same-age peers and better than the grammar-matched children. The contrast between the results of the two motor procedural learning tasks revealed a unique contribution of sequence to poor motor procedural learning in SLI.

In the study of Tomblin *et al*., although teenagers with SLI did worse than controls, a significant re-bound in RT was seen in both typically developing and SLI groups; in contrast, we did not find a significant re-bound in RTs in either the SLI group or the grammar-matched group. Furthermore, the magnitude of the re-bound in RTs of the typically developing adolescents (mean age = 14.76) in Tomblin *et al*.’s study was larger than that of the 7- to 11-year-old (age-matched) control group in the current study. The discrepancy in findings might be due to age differences of the participants in the two studies. The 7- to 11-year-old typically developing children had started to show evidence of motor sequence learning, though the magnitude of such learning was smaller than had been observed in older children. On the contrary, the children with SLI, like the younger typically developing children, showed no evidence of learning the repeated sequence.

On the pursuit rotor task, we also tested retention of learning after an interval of around 2 weeks, and found that children with SLI were able to maintain the skills learned from the first session to the same level as the age-matched children. This finding contrasts with results from Hedenius *et al*. (2011), who reported poor long-term learning of SRT in children with SLI. Future studies should directly compare long-term retention of different procedural learning tasks in the same children with SLI; the pattern of results to date provides converging evidence that poor long-term procedural learning might be tied to learning sequence-specific information.

The idea that procedural learning is not a unitary construct is not new (e.g. Squire, 1987). The convention that pursuit rotor, SRT and several other paradigms are viewed as standard measures of procedural memory comes from neuropsychological studies in which these tasks often elicited the same results from patients with a similar neuropathology. Previous studies on neural imaging and patients have converged on the finding of the importance of the striatum and frontal regions in processing sequential information in SRT tasks (Doyon, Owen, Petrides, Sziklas & Evans, 1996; Ferraro, Balota & Connor, 1993; Grafton, Hazeltine & Ivry, 1995; Knopman & Nissen, 1991; Pascual-Leone, Grafman, Clark & Stewart, 1993; Peigneux, Maquet, Meulemans, Destrebecqz, Laureys, Degueldre, Delfiore, Aerts, Luxen, Franck, Van der Linden & Cleermans, 2000; Poldrack, Sabb, Foerde, Sabrina, Asarnow, Bookheimer & Knowlton, 2005; Rauch, Savage, Brown, Curran, Alpert, Kendrick, Fischman & Kosslyn, 1995; Rauch, Whalen, Savage, Curran, Kendrick, Brown, Bush, Breiter & Rosen, 1997; Rauch, Whalen, Curran, McInemey, Heckers & Savage, 1998; Willingham & Koroshetz, 1993). Some studies also reported striatal involvement (e.g. putamen) in pursuit rotor tasks (Grafton *et al*., 1992; Grafton *et al*., 1994; Sarazin *et al*., 2002; Schmidtke *et al*., 2002). However, few studies have directly compared the neuroanatomical and neurophysiological basis involved in learning the two motor procedural tasks. In addition, much less is known about the neurological basis involved in Hebb repetition learning. One interesting direction of future studies building on the current findings would be to gain better understanding of the structural and functional differences between typically developing children and children with language impairments when performing these tasks. This would not only further our understanding of the behavioural differences observed in these tasks, but would also provide insight into individual differences in language abilities.

In the current study, the pattern of results on the Hebb learning task was similar to that observed on the SRT task: children with SLI were impaired relative to same-age controls and showed similar performance to the younger, grammar-matched children. The observed group differences cannot be explained by individual differences in short-term memory, as an adaptive list length based on each child’s word span was used. Furthermore, the group that did best, the age-matched controls, had to remember longer lists on average. Another factor that could affect performance is familiarity with the vocabulary used in the test, but it is unlikely that this was a significant contributor to the results because we used highly familiar words. It is also noteworthy that a closely similar pattern of results was found for the nonverbal SRT task, where such factors would not apply.

Tomblin *et al*. (2007) suggested that the association between verbal and motor sequence learning might reflect a domain-general learning system that is capable of extracting structures in complex input. This is consistent with the accumulating evidence in the literature that similar serial-position mechanisms operate across different modalities regardless of which type of stimuli are being processed (Depoorter & Vandierendonck, 2009; Guérard & Tremblay, 2008; Jones, Farrand, Stuart & Morris, 1995; Parmentier, Elford & Maybery, 2005; Smyth, Hay, Hitch & Horton, 2005; Smyth & Scholey, 1996; Ward, Avons & Melling, 2005; cf. Conway & Christiansen, 2005, 2006). In the current study, we found that performance in the motor sequence learning task was associated with performance in the verbal sequence learning task, consistent with the view of a domain-general learning system for sequential information.

As well as finding a different pattern of results for sequential vs. nonsequential procedural learning, we also found close similarities between performance of children with SLI on sequential tasks and that of younger typically developing children who were matched on a test of grammatical comprehension. This raises the question of whether the kind of sequential learning tested in these tasks is a key component of language learning. The verbal and motor sequence learning tasks used in the current study involved simple adjacent learning of deterministic sequence (i.e. the same sequence being repeated), whereas grammatical learning of syntactic structures additionally involves learning non-adjacent dependencies from exposure to probabilistic sequences (Gomez, 2002; Hsu & Bishop, 2010; Tallerman, Newmeyer, Bickerton, Bouchard, Kaan & Rizzi, 2009). Tomblin *et al*. (2007) argued that the sequence-specific learning tapped by the SRT task is likely also to be involved in extraction of higher-order relationships such as graded probabilistic relationships or chunks of sequences (Cleeremans & McClelland, 1991; Curran & Keele, 1993; Stadler, 1995), but it would be of interest to look explicitly at this issue in future studies of SLI.

One core argument of the procedural deficit hypothesis is that children with SLI are disproportionately impaired in grammar and are relatively spared in vocabulary. Although the current finding of similar SRT performance in the SLI and grammar-matched groups seems to support the hypothesis, the children with SLI in the current study did not show significantly superior vocabulary size than the grammar-matched children (see Table 1). In fact, Lum and colleagues (2010, 2011; Lum & Bleses, 2012) have also found evidence of poor declarative learning in children with SLI. It is true that SLI constitutes a heterogeneous group and that the language conditions described by the procedural deficit hypothesis would capture the language profile in at least some children with SLI. However, it would be over-simplistic to conclude that poor procedural learning is the only, or even the major, deficit responsible for the language impairments typically seen in SLI. In natural learning contexts, procedural and declarative systems will interact, as children learn from language input that contains grammatical, phonological and semantic information. Deficits affecting nonverbal as well as verbal learning of sequential information appear to be a robust finding in SLI, but we need to remember that such learning does not operate in isolation; interaction between procedural and declarative systems needs to be taken into account when considering language development in both typical and atypical contexts.

## Appendix

### Sample lists of the Hebb repetition task (length = 4)

1. *dog bus sock comb*
2. chair soap ball cup
3. knife dress hat cot
4. *dog bus sock comb*
5. lion watch mug toy
6. shoe pen door book
7. *dog bus sock comb*
8. leg cat jug tree
9. car juice owl star
10. *dog bus sock comb*
11. arm toast fork key
12. deer moon box tea
13. *dog bus sock comb*
